# Arachidonic Acid Metabolite as a Novel Therapeutic Target in Breast Cancer Metastasis

**DOI:** 10.3390/ijms18122661

**Published:** 2017-12-08

**Authors:** Thaiz F. Borin, Kartik Angara, Mohammad H. Rashid, Bhagelu R. Achyut, Ali S. Arbab

**Affiliations:** Tumor Angiogenesis Laboratory, Georgia Cancer Center, Department of Biochemistry and Molecular Biology, Augusta University, Augusta, GA 30912, USA; kangara@augusta.edu (K.A.); mrashid@augusta.edu (M.H.R.); bachyut@augusta.edu (B.R.A.); aarbab@augusta.edu (A.S.A.)

**Keywords:** breast cancer metastasis, cytochrome P450, 20-HETE

## Abstract

Metastatic breast cancer (BC) (also referred to as stage IV) spreads beyond the breast to the bones, lungs, liver, or brain and is a major contributor to the deaths of cancer patients. Interestingly, metastasis is a result of stroma-coordinated hallmarks such as invasion and migration of the tumor cells from the primary niche, regrowth of the invading tumor cells in the distant organs, proliferation, vascularization, and immune suppression. Targeted therapies, when used as monotherapies or combination therapies, have shown limited success in decreasing the established metastatic growth and improving survival. Thus, novel therapeutic targets are warranted to improve the metastasis outcomes. We have been actively investigating the cytochrome P450 4 (CYP4) family of enzymes that can biosynthesize 20-hydroxyeicosatetraenoic acid (20-HETE), an important signaling eicosanoid involved in the regulation of vascular tone and angiogenesis. We have shown that 20-HETE can activate several intracellular protein kinases, pro-inflammatory mediators, and chemokines in cancer. This review article is focused on understanding the role of the arachidonic acid metabolic pathway in BC metastasis with an emphasis on 20-HETE as a novel therapeutic target to decrease BC metastasis. We have discussed all the significant investigational mechanisms and put forward studies showing how 20-HETE can promote angiogenesis and metastasis, and how its inhibition could affect the metastatic niches. Potential adjuvant therapies targeting the tumor microenvironment showing anti-tumor properties against BC and its lung metastasis are discussed at the end. This review will highlight the importance of exploring tumor-inherent and stromal-inherent metabolic pathways in the development of novel therapeutics for treating BC metastasis.

## 1. Introduction

Breast cancer (BC) is composed of multiple subtypes with distinct morphologies and clinical implications. Histologically, BC can be classified according to tissue morphology into ductal and tubular types, which are further divided into benign or invasive subtypes [1]. Additionally, four major molecular categories are used to classify BC according to their steroid hormone receptor status and the presence or absence of the human epidermal growth factor receptor 2 (HER2). The luminal A subtype is characterized by the presence of an estrogen receptor (ER) and/or progesterone receptor (PR); luminal B is ER+ and/or PR+ and HER2+. The HER2-enriched tumors are positive for HER2+ expression and negative for both steroid hormone receptors. The basal-like or triple-negative breast cancer (TNBC) subtype is negative for all three receptors. Other molecular classifications can be used to complement distinctive gene and protein expression signatures such as claudin (low or high), Ki67 rates, or mesenchymal and epithelial marker status to predict personalized treatment and prognosis for BC patients. The percentage of each subtype presenting clinically, as well as their associated prognosis, is summarized in Table 1.

Remarkably, these molecular subtypes are strongly associated with survival: luminal A tumors have the most favorable prognosis; luminal B, HER2-positive, and basal-like tumors are associated with the shortest relapse-free and overall survival rates [13]. Molecular subtypes also predict treatment response, with HER2-positive and TNBC tumors being more sensitive to preoperative chemotherapy than the luminal tumors [14]. BC has a propensity for distant metastasis to the bones, lungs, brain, and liver [15,16,17,18]. Bone metastasis is the first most common site of distant spread, having the longest median survival duration of about two to five years. However, patients with brain metastasis (BM) have the shortest survival of around four to seven months [16,19,20]. It has been reported that up to 15–30% of metastatic BC patients will eventually develop BM during the course of the disease [21,22]. Luminal A and B subtypes have a low risk of BM, of 2–9% and 4–10%, respectively [20,23,24], while HER2-enriched and TNBCs exhibited high rates of brain, lung, bone, and distant nodal metastases of 15–30%, 20–30%, 10–25%, and 17.2%, respectively [7,10,11]. Not all BC cells in primary tumors possess metastatic potential, and only a small subpopulation of cells can home to distant tissues or organs [25]. Metastasis remains one of the major causes of mortality in BC; however, no standardized therapy is available. Since the outcomes of tumor cell-targeted therapies are poor, tumor-associated stroma could be targeted to inhibit BC metastasis.

Interestingly, BC has been shown to thrive in the tumor microenvironment (TME), which consists of a pro-tumorigenic pathological immunosuppressive niche not only for BC cells themselves, but also for a significant amount of surrounding stroma and tumor-associated cells. Diverse components of the BC microenvironment, such as suppressive immune cells, re-programmed fibroblast cells, pathological neovascular structures, altered extracellular matrix, and certain soluble factors, synergistically impede an effective anti-tumor response and promote BC progression and metastasis [26]. BC cells recruit tumor infiltrated lymphocytes such as T-regulatory cells, myeloid-derived suppressor cells (MDSCs), and M2-macrophages to induce a pro-tumorigenic environment that attenuates anti-tumor immunity [27,28]. Aberrant expansion and accumulation of MDSCs have been extensively reported in BC. MDSCs are a heterogeneous population of immature granulocytes, macrophages, and dendritic cells [29] that are recruited to the primary tumor as well as the metastatic site and play a crucial role in inhibiting innate and adaptive immune responses by suppressing CD4+ T-cells, CD8+ T-cells, and natural killer (NK) cells [30,31,32]. In clinical scenarios, circulating MDSCs have been shown to have a positive correlation with BC stage and metastatic tumor burden [33]. In addition, increased numbers of MDSCs are correlated with the rate of recurrence and metastasis of BC [28,34,35,36,37].

Another important pro-tumorigenic myeloid subset in the TME are the macrophages, which are either residents or derived from the spleen or bone marrow [38,39]. Tumor-associated macrophages (TAMs) can be present as an M1 subtype that produces type 1 pro-inflammatory cytokines promoting the anti-tumorigenic role and an M2 subtype that produces type 2 anti-inflammatory cytokines that facilitate a pro-tumorigenic environment [40]. In the hypoxic tumor core, M1 macrophages polarize and switch phenotypes to M2 macrophages to promote pathological angiogenesis, thereby making the tumor more aggressive and invasive [41]. Furthermore, a metastatic subpopulation of TAMs was observed in a mouse model to promote the extravasation, invasion, and colonization of BC cells in the metastatic site [38,42]. Current therapies, including chemotherapy and targeted therapies, are failing due to the immunosuppression caused largely by MDSCs and TAMs in the primary tumor or the metastatic sites [43]. It is therefore important to understand the mechanisms causing this therapy resistance, tumor relapse, and refractoriness.

Recently, we observed that targeting the arachidonic acid (AA) pathway by inhibiting the synthesis of 20-hydroxy-eicosatetraenoic acid (20-HETE) resulted in the decreased migration and invasion of metastatic BC cells. In addition, in the same study, we found a synergistic reduction of the granulocytic MDSC (g-MDSCs: CD11b+Ly6G+) populations in the metastatic niches [44]. In our previous studies, we have been able to demonstrate a decrease in the levels of pro-angiogenic factors that are responsible for the communication between tumor cells and the microenvironment with a selective 20-HETE inhibitor, *N*-hydroxy-*N*′-(4-butyl-2 methyl phenyl) formamidine (HET0016), alone or in combination with anti-angiogenic therapies. Anti-20-HETE therapy was able to decrease breast and glioma tumor sizes [45,46]. Interestingly, the anti-AA pathway therapy was more effective at reducing tumor volume, the level of pro-angiogenic factors, and extent of metastasis than the antiangiogenic therapies used. It is therefore important for researchers to focus on and understand the role of metabolic pathways in tumors and their interplay with the stroma. In the current review, we have focused exclusively on the AA-20-HETE pathway and its implications in modeling the TME.

## 2. Arachidonic Acid Metabolism

**Figure 1:**
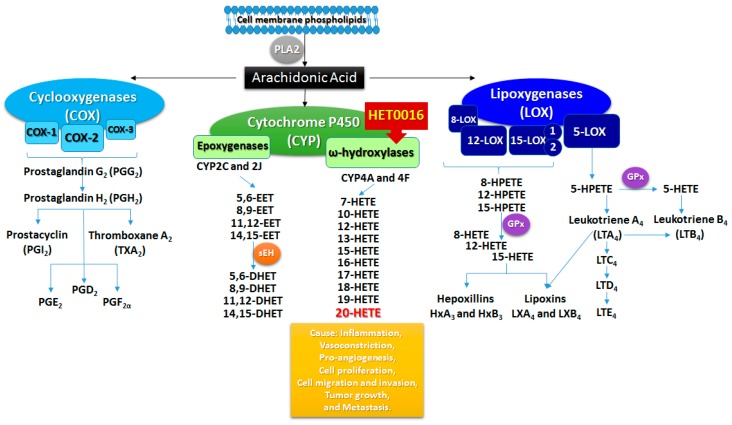
Schematic representation of phospholipid-arachidonic acid metabolites produced via the major enzymes cyclooxygenase (COX), lipoxygenase (LOX), and cytochrome P450 (CYP4A). CYP4A produced 20-hydroxy-eicosatetraenoic acids (20-HETE) metabolite, which is known to promote tumor growth. Legend: phospholipase A2 (PLA2); epoxy-eicosatrienoic acids—(EETs); epoxide hydrolase (sEH); dihydroxy-eicosatrienoic acids (DHETs); hydroperoxy-eicosatetraenoic acid (HPETE); glutathione peroxidase (GPx).

AA is a polyunsaturated ω-6 fatty acid present in the phospholipids of cell membranes, which is abundant in the brain, muscles, and liver [47,48,49]. The main precursors (fatty acids) of AA are obtained through the diet and its synthesis involves the expression of enzymes regulated in situ [50] after the activation of phospholipase A2 (PLA2) by neuroeffectors such as norepinephrine, angiotensin II, and bradykinin [51]. AA produces different biologically active metabolites through three different enzymatic pathways (Figure 1): the cyclooxygenase (COX), lipoxygenase (LOX), and cytochrome P450 (CYP) pathways [52]. These metabolic products can modulate renal, pulmonary, and cardiovascular functions, vascular tone, and inflammatory responses as paracrine factors and second messengers [53,54,55]. The COX pathway has two main enzymes, COX-1 and COX-2, which are critical in the regulation of inflammation and tissue homeostasis. Both COX-1 and COX-2 enzymes act on the AA synthesized from the cell membrane phospholipid by PLA2, and then metabolize AA into an intermediate prostaglandin (PG) H2 through PGG_2_ (Figure 1) [56,57]. PGH_2_ is an unstable endoperoxide that is catalyzed by specific synthases and generates five major prostanoids such as PGD_2_, PGE_2_, PGF_2α_, PGI_2_ (prostacyclin), and thromboxane A_2_ that have an important role in cancer-associated inflammation, tumor progression, and metastasis [57,58]. COX-1 is constitutively expressed in almost all tissues and inflammatory cells, and generates PGs that control homeostasis [56,57]. COX-2 is transiently and highly expressed in response to growth factors and endotoxins and is often involved in inflammation, cell proliferation, and differentiation [56]. The COX-2 pathway has also been targeted in many cancer studies, including colon cancer, colorectal cancer, breast cancer, gliomas, prostate cancer, esophageal carcinoma, pancreatic cancer, and lung carcinoma, due to its increased expression and correlation with the reduction of survival rates in cancer patients [56,57,58]. It is already known that most cancerous tissues show signs of inflammation in the pre-cancer stages, and chronic stimulation by innate immune cells, cytokines, and chemokines lead to malignant transformation and tumor progression [58]. COX-2 inhibitors have been extensively studied through their properties to inhibit tumor growth by suppressing inflammation and angiogenesis. However, patients treated with celecoxib, a COX-2 inhibitor, in clinical trials demonstrated gastrointestinal complications, a higher risk of cardiovascular toxicity, and death [58]. Even then, the toxicities of COX-2 inhibitors do not exclude the importance of this treatment as an adjuvant in cancer therapy.

A third isoform of COX enzymes has been identified primarily in canine samples and then confirmed in human tissue. COX-3 contains all of the COX-1 protein information, except for the retained intron sequence that alters its enzymatic properties, significantly generating PGE_2_ [59]. The functional COX-3 biosynthesis is an important concept that can help to explain the prostaglandin-independent, anti-inflammatory actions previously attributed to the reactivation of COX-2 activity at a later stage in enhancing inflammatory resolution [60,61]. However, further studies showing whether there is a unique human COX-3 that acts independently of COX-1 and COX-2 and to determine the role of COX-3 in tumor growth and development are still lacking.

The LOX pathway produces different leukotrienes (LTs), lipoxins (LXs), hepoxillins (HOs), and hydroxy-eicosatetraenoic acids (HETEs), causing inflammation, allergic reactions, bronchoconstriction, and vasoconstriction (Figure 1). The principal lipoxygenases expressed in humans are 5-lipoxygenase (5-LOX), 8-lipoxygenase (8-LOX), 12-lipoxygenase (12-LOX), and 15-lipoxygenase (15-LOX) type 1 and 2. In general, the LOX pathway catalyzes the oxygenation of AA into hydroperoxy-eicosatetraenoic acid (HPETE) and converts HPETEs to LTs, LXs, Hos, and HETEs through the reduction of HPETE to HETE [62]. The four distinct enzymes insert oxygen at the specific carbons 5, 8, 12, or 15 of AA, generating 5-, 8-, 12-, or 15-HPETE, which can be further reduced by glutathione peroxidase (GPx) to the hydroxy forms (5-, 8-, 12-, 15-HETE), respectively [63]. The 5-LOX pathway synthesizes the key pro-inflammatory LT mediators such as leukotriene A4 (LTA_4_) and leukotriene B4 (LTB_4_) [58]. LTA_4_ is an unstable LT that can be converted into LTB_4_ or cysteinyl LTs (LTC_4_, LTD_4_, and LTE_4_) [57]. High levels of LTB_4_, the main product of the 5-LOX pathway, were found in prostate cancer samples [64], and its receptors were found to be overexpressed in gastric cancer compared to normal tissue [65]. Interestingly, an LTB_4_ receptor antagonist in combination with chemotherapy was able to decrease tumor growth and metastasis in vitro and in vivo in colon cancer and pancreatic cancer models [66]. However, this combination did not change the survival rates in pancreatic or lung cancer clinical trials [67]. 8-LOX and its products 8-HPETE and 8-HETE are expressed in skin, mainly after irritation, but their importance in tumorigenesis remains unclear and poorly reported [63].

12-LOX also has a critical role in tumor angiogenesis, motility, invasion, and metastasis [68]. 12-LOX is the main human 12-HETE-generating enzyme and can synthesize 12S-HPETE through AA or either 12S- or 15S-HPETE through linoleic acid metabolism [57,63]. Three isoforms of the 12-LOX enzyme have been identified, including the leukocyte and platelet type (named as S) and epidermal type (named as R), expressed in various types of cells such as leukocytes, platelets, smooth muscle cells, endothelial cells, and keratinocytes [57]. Evidence shows that both the leukocyte- and platelet-types of 12-LOXs have been found in cancer tissues such as melanoma, prostate, and epidermal cancers and promote cell proliferation and survival [69]. 12-LOX inhibition can decrease the proliferation and induce apoptosis in human gastric cancer cells, prostate cancer cells, and carcinosarcoma cells [56,70,71]. 12S-LOX also converts AA to HOs by reducing 12S-HETE into 8-hydroxy-11,12-epoxy-eicosatetraenoic acid (HxA_3_) and 10-hydroxy-11,12-epoxy-eicosatrienoic acid (HxB_3_) or by its isomerization [72]. HOs exhibit vast biological activities including the stimulation of insulin secretion by glucose induction, an increase of intracellular calcium levels in pancreatic islets cells, and the induction of hyperpolarization of the membrane potential in neurons [72].

15-LOX is subdivided into two isoforms, 15-LOX-1 and 15-LOX-2. They are widely distributed in the tissues and mainly expressed in reticulocytes, eosinophils, pulmonary epithelial cells, and macrophages [57]. 15-LOX has an ambiguous activity, being pro- or anti-tumorigenic depending on its subtype. 15-LOX-1 metabolizes linoleic acid to 3-hydroxy-octadecadienoic acid (13S-HODE) and metabolizes AA to 15S-HETE. However, 15-LOX-2 mainly converts AA to 15S-HETE [57,63].

Lipoxins are trihydroxy-eicosatetraenoic acids derived from three different pathways of AA metabolism. They can be synthesized by the platelet–leukocyte interaction that involves the production of LTA_4_ by 5-LOX in neutrophils and its conversion to lipoxin A4 (LXA_4_) and lipoxin B4 (LXB_4_) by 12-LOX in platelets upon their adherence to leukocytes. Without this interaction the production of LXs does not happen [73]. The generation of LXs can also be achieved through the oxygenation of AA in the presence of 15-LOX, generating 15S-HPETE, which serves as a substrate for 5-LOX, or through COX-2 acetylation, which generates COX-2-derived HETE and is converted by 5-LOX to 15-epi-lipoxin A4, also known as aspirin-triggered lipoxin (ATL) and 15 epi-lipoxin B4 [73,74]. Lipoxins and epi-lipoxins show anti-inflammatory effects through signals engendered by binding to G protein-coupled lipoxin A4 receptor (ALX)/formyl peptide receptor (FPR2) [74]. Lipoxins have been shown to downregulate NFκB expression and could be used as a potential treatment for several cancer types [74]. LXA_4_ can decrease cell proliferation, inhibits cell invasion, and suppresses tumor growth, exhibiting anti-inflammatory properties in cancer cells [58]. Since LXs can target a variety of inflammatory and angiogenic molecules, inhibitors of LTA_4_ hydrolase could be potentially used in a combination therapy along with standard chemotherapeutic drugs to treat cancer [58,74]. Further in vivo studies are required to corroborate the idea of whether LXs could be used as an adjuvant in preventing cancer progression.

The COX and LOX pathways represent two major routes of AA metabolism that controls the biosynthesis and activity of LTs, LXs, HOs, and HETEs or intermediary products such as HPETEs. These products can act as effectors in inflammatory responses or activate the production of second messengers such as reactive oxygen species (ROS) through interaction with cognate G protein-coupled cell-surface receptors or nuclear receptors such as peroxisome proliferator activated receptors (PPARs) [75]. CYP enzymes require nicotinamide adenine dinucleotide phosphate (NADPH) reductase and CYPb5 as cofactors and are a major source of superoxide ions, releasing a significant amount of oxygen radicals in the vasculature, which makes the metabolism of AA by CYP enzymes an important contributor to oxidative stress [53]. Oxidative stress activates a host of pro-inflammatory cytokines and chemokines such as TNF-α and IL-8 and adhesion molecules such as ICAM-1, E-selectin, or P-selectin. The metabolic end products generated as a result of the peroxidation of lipids also serve as potent chemoattractants for inflammatory cells [76]. Two key inducible cytochrome P450 enzymes, CYP2E1 and CYP4A, involved in lipid peroxidation function complementarily, and this may lead to interactions in the regulation of these enzymes [77,78]. CYP2E1 and CYP4A have been demonstrated to function as leaky enzymes that are capable of undergoing “futile cycling”. During this process, even in the absence of a substrate, these enzymes are capable of producing ROS such as superoxide anions, hydroxyl radicals, and hydrogen peroxides [79,80,81]. Reduction in the levels of a key antioxidant such as glutathione (GSH) by inhibiting GSH synthesis with buthionine sulfoximine (BSO) dramatically upregulated the AA levels, causing toxicity. Overexpression of the antioxidant enzyme catalase countered the pro-oxidant activity of CY2E1 [82]. In response to cell mediated injury by AA metabolism, especially in CYP2E1-overexpressing cells, antioxidant molecules such as GSH were high due to the upregulation of γ-glutamylcysteine synthetase [83], GSH S-transferases, and catalase [84]. The contributions of these pathways in cancer development and their interaction and deregulation are still open for discussion. More extensive investigation is needed to delineate how COX and LOX inhibitors could be more effective in decreasing tumor growth and metastasis. In this review, our main focus is the CYP pathway and its metabolites in relation to various aspects of inflammation and cancer.

The cytochrome P450 (*CYP*) gene family consists of a complex 18 gene families that encode more than 103 functional genes in mice and 57 genes in humans [85,86]. The *CYP2*, *CYP3*, and *CYP4* families encode more genes than the remaining 15 families in human as well as in rodent genomes [86]. The majority of the genes found in the *CYP1*, *CYP2*, *CYP3*, and *CYP4* families encode enzymes involved in eicosanoid metabolism and are inducible by diet, chemical inducers, drugs, pheromones, and other factors [86]. Their function is predominantly in the detoxification of drugs, toxic compounds, chemotherapies, xenobiotics, and products of endogenous metabolism such as bilirubin in the liver [85,86]. The *CYP2* and *CYP3* families are the most redundant, mutated, or defective in one or more genes compared to the other 16 gene families that might be responsible for the CYP-related diseases that will be directly involved in their critical life functions [86].

The CYP pathway is an enzymatic pathway divided into ω-hydroxylase and epoxygenase pathways that use AA as a substrate to produce eicosanoids. Derivatives of the ω-hydroxylase pathway (HETEs) cause inflammation, vasoconstriction, vascular remodeling, and cellular proliferation. Metabolites of the epoxygenase pathways (epoxy-eicosatrienoic acids—EETs) resolve inflammation and cause vasodilation, the protection of cardiac function, and cell proliferation [85,87,88]. In mammalian cells, the most studied and effective subfamily to produce 20-HETE is CYP4A [53]. In rats, there are four isoforms identified: CYP4A1, CYP4A2, CYP4A3, and CYP4A8 [89]. These isoforms share 66–98% homology and common catalytic activity and are expressed in the liver, kidney, and brain [90]. CYP4A1 has the highest catalytic efficiency to convert AA into 20-HETE, followed by CYP4A2 and CYP4A3; however, CYP4A8 did not catalyze AA or linoleic acid [91]. In mice, CYP4A10, CYP4A12a, CYP4A12b, and CYP4A14 are the principal isoforms that catalyze AA ω-hydroxylation to 20-HETE [92]. CYP4A10 has a lower catalytic activity for 20-HETE production than the CYP4A12 isoforms. CYP4A12a and CYP4A12b have similar hydroxylase activity, constituting the major source of 20-HETE synthesis [92]. Particularly, in addition to the CYP4A enzymes, the CYP4F isoforms are also significant for 20-HETE production [90]. In humans, the isoforms CYP4A11, CYP4A22, CYP4F2, and CYP4F3 are the most important in the production of 20-HETE, predominantly CYP4F2, followed by CYP4A11 [93]. The isoforms and their species-specific expression are summarized in Table 2.

The ω-hydroxylases from the CYP family 4, subfamily A (*CYP4A*), and F (*CYP4F*) genes convert AA into 7-, 10-, 12-, 13-, 15-, 16-, 17-, 18-, 19-, and 20-HETEs, and the epoxygenases mainly encoded by the *CYP* family *2* subfamilies *C* and *J* genes generate 5,6-EET, 8,9-EET, 11,12-EET, and 14,15-EET, which will be further metabolized into the less active dihydroxy-eicosatrienoic acids (DHETs) through epoxide hydrolase (sEH) [58,85]. All four EETs and their metabolite DHET can act as a long-chain of fatty acids and stimulate the peroxisome proliferator response element to bind to PPAR [85]. 20-HETE is the principal pro-inflammatory metabolite produced by the ω-hydroxylase enzymes and regulates vascular remodeling and neovascularization under ischemic or hypoxic conditions [94,95,96]. 20-HETE synthesis can be controlled through the activation of calcium/calmodulin-dependent kinase II and mitogen-activated protein kinase (MAPK) in smooth muscle cells [97]. 20-HETE can be incorporated into endothelial lipids through a coenzyme A-dependent process and is further metabolized by ω-oxidation or β-oxidation to 20-carboxy-arachidonic acid (20-COOH-AA) [98]. The metabolism of 20-HETE can also be regulated through COX-mediated pathways into 20-hydroxy-prostaglandin G2 and H2 [99]. 20-HETE stimulates the activation of the nuclear factor κB (NFκB) and MAPK/ERK pathways, mediating pro-inflammatory effects, and also has an important role in epidermal growth factor (EGF), hypoxia-inducible factor (HIF), and vascular endothelial growth factor (VEGF) activation, showing pro-angiogenic effects and the stimulation of endothelial cell proliferation, migration, and cell survival [94,100]. Recently, molecular studies highlighting the relationship between aberrant AA metabolism through 20-HETE downstream signaling pathway activation and carcinogenesis have provided novel molecular targets for cancer chemoprevention and treatments.

## 3. Cytochrome P450 Mechanisms in Obesity and Breast Cancer

Dysregulated energy metabolism is already known to be a hallmark of cancer [101]. One of the main examples of dysregulated energy metabolism is obesity, which has been associated with high levels of aromatase in breast tumors and undifferentiated adipose tissue [102]. Aromatase is a CYP19 enzyme responsible for the critical steps in the synthesis of estrogens [103] that are most related to breast cancer risks. Several drugs, in particular anti-diabetic drugs, have shown effects in decreasing tumor growth, breast cancer recurrence, and metastasis [102,104]. Metformin, for example, can inhibit aromatase expression via 5′ AMP-activated protein kinase (AMPK) in breast adipose stromal cells [105]. In breast cancer, adipose tissue provides structural and paracrine support for tumor development and growth. In addition to adipose tissue in the breast, other stromal cells can provide crucial metabolites through the CYP-mediated lipid peroxidation pathway to favor tumor growth and the production of pro-tumorigenic eicosanoids.

AA and its metabolites have been strongly implicated in the pathogenesis of obesity and related complications in peripheral tissues and organs owing to their ability to provide fatty acids for the production of pro-inflammatory cytokines [106]. The constituent expression of all the molecular players crucial for the 5-LOX pathway to generate leukotrienes and the receptors (two LTB_4_ [BLT1 and BLT2] receptors and two cysteinyl LT [CysLT-R1 and CysLT-R2]) receptors in the adipocyte and the stromal vascular fraction highlight the importance of this pathway in obesity. LTB_4_ signaling plays a crucial role in mediating the differentiation of preadipocytes to mature adipocytes, and 5-LOX-derived leukotrienes are elevated in the obese adipose tissue [107,108,109,110,111,112]. 5-LOX has also been indicated in the modulation of lipid metabolism to provide free fatty acids as substrates for the production of pro-inflammatory eicosanoids [110]. In our studies, we have demonstrated that HET0016 is a selective CYP4A and CYP4F ω-hydroxylase inhibitor that does not have any possible effects on the 5-LOX signaling pathway to control obesity. However, a recent study by Park et al. [113] showed the effects of the inhibition of CYP4A enzyme activity in type 2 diabetes mellitus (T2DM) in obese mice. The authors reported that obesity is one of the important causes of elevated endoplasmic reticulum stress in obese mice and identified 54 novel CYP4A enzyme isoforms that were upregulated in obesity-induced T2DM in the *db*/*db* mice model, of which CYP4A10 and CYP4A14 levels were significantly upregulated [113]. Since HET0016 has specificity for the inhibition of CYP4A enzymes, it can be strongly hypothesized that the effects of HET0016 can be observed in animals. Animals fed with a high-fat diet and treated with HET0016 for 12 weeks presented a significantly decreased body weight and total fat-pad mass, and improved glucose tolerance and insulin sensitivity compared with animals that were not fed the high-fat diet [113]. The fasting blood glucose concentration in obese animals was comparable to the levels observed in normal-diet-fed animals [113]. We did not find any evidence of studies in humans. The specificity of CYP4A for ω-oxidation facilitates the degradation of long-chain fatty acids, therefore providing a secondary metabolic pathway for the metabolism of fatty acids when levels of these substrates increase during the physiological processes of lipolysis and hepatic fatty acid uptake. It would be fascinating to investigate the other mechanisms involved in the inhibition of the CYP4A-mediated control of obesity in these animals. However, the current review is focused on presenting evidence as to how the inhibition of 20-HETE, an AA metabolite, might be a novel therapeutic target in BC metastasis.

## 4. Arachidonic Acid Pathway and 20-HETE in Primary Tumors and Metastasis

In tumors, the CYP4A/20-HETE axis promotes inflammation, endothelial cell migration, and neovascularization [114,115,116,117,118,119]. When *N*-hydroxy-*N*′-(4-butyl-2 methyl phenyl) formamidine (HET0016), a highly selective inhibitor of 20-HETE synthesis, was used alone in tumor-bearing animals, a decrease in tumor growth was observed via impaired tumor neovascularization [44,45,46,120]. HET0016 is also shown to decrease MAPK signaling, pSTAT1, EGFR, and HIF-1α in glioblastoma (GBM) tumor lysates [121]. When the expression of different pro- and anti-angiogenic factors and inflammatory cytokines in the tumor lysates were analyzed, there were significant changes following HET0016 treatments compared to that of vehicle-treated tumors [46,121]. When the extravascular extracellular space (EES), different vascular parameters, and neovascularization were examined, HET0016 treatment significantly decreased EES, tumor blood volume, permeability, and neovascularization [46,121]. We also reported that HET0016 decreased vascular mimicry, a phenomenon where tumor cells make blood vessel-like structures [120,122]. We found that the CYP4A/20-HETE axis plays a critical role in metastasis in a syngeneic model of BC-mediated pulmonary metastasis. Targeting 20-HETE production also decreased pulmonary metastasis in an aggressive BC model [44]. When applied at the pre-metastatic stage, HET0016 significantly decreased pulmonary metastatic growth through decreasing a survival pathway (p-AKT), inflammation pathway (canonical NFκB signaling), migration pathway (matrix metalloproteinase-2 and -9, MMP2 and 9), and mesenchymal cancer stem cell markers (CD44 and *N*-cadherin) in the metastatic lung niche [44]. In cancer studies, 20-HETE mediated effects have been studied in the context of tumor cells and endothelial progenitor cells (EPCs). Investigations regarding the contribution of tumor-associated stromal cells such as myeloid cells are rare. The following sections will discuss the role of stromal and myeloid cell-mediated 20-HETE production and its effects on tumor growth and metastasis.

## 5. Role of 20-HETE in Stromal Cells and Tumor Cells

Initially, the entirety of cancer research was focused on the idea that tumor growth and metastasis were tumor-cell inherent/autonomously driven by mutations arising in these tumor cells to meet with their metabolic and nutritional needs. This dogma has been refuted to incorporate the idea of a “tumor microenvironment” comprised of stromal cells such as endothelial cells, fibroblasts, and pericytes and also infiltrating myeloid cells such as monocytes, macrophages, and MDSCs to favor tumor growth and metastasis [123,124,125,126,127]. The non-tumor cell-dependent contribution to tumor growth and progression has gained immense popularity, thanks to the landmark hypothesis of tumor angiogenesis proposed by Folkman in 1971 [128]. Tumor neovascularization has now been extended to include vasculogenesis, intussusception, tumor cell transdifferentiation to endothelial phenotypes, and vascular mimicry [129,130].

The ω-hydroxylation of therapeutic drugs, as well as endogenous compounds, e.g., fatty acids, by the CYP4 family members functions to metabolically activate and further eliminate these compounds. Eicosanoids, derived from AA, are key substrates of this cytochrome P450-dependent oxidation reaction. Human CYP4 enzymes such as CYP4A11, CYP4F2, and CYP4F3B, hydroxylate AA at the omega position to form 20-HETE. 20-HETE has already been shown to have hallmark effects in tumor progression, angiogenesis, and inflammatory processes associated with tumor growth and metastasis. The processes of tumor-associated angiogenesis and inflammation driving the immunosuppression go hand-in-hand and thus exert significant influence on tumor growth and metastasis. The pro-inflammatory cytokines and pro-growth factors work to promote tumor growth; the tumor thereby releases various chemokines/cytokines that promote the recruitment of MDSCs, neutrophils, macrophages, and other myeloid cells to facilitate the development of an immunosuppressive niche conducive to tumor growth [131,132,133].

**Figure 2:**
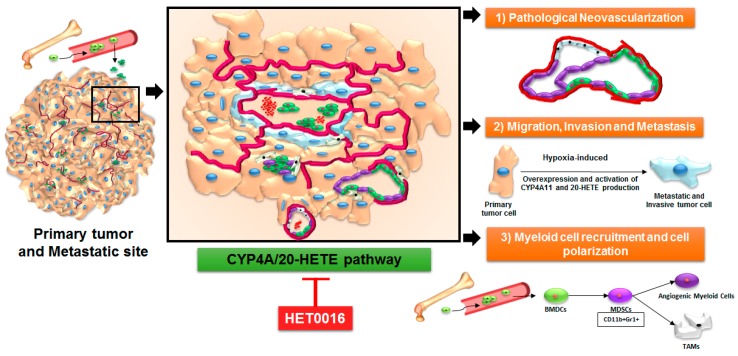
Schematic representation of the involvement of the CYP4A/20-HETE pathway in the primary tumor microenvironment and its potential metastatic site. (**1**) The CYP4A/20-HETE pathway is overexpressed in myeloid-derived suppressor cells (MDSCs) recruited to the primary tumor and in the tumor-associated stroma cells, promoting polarization to a g-MDSC phenotype; (**2**) The CYP4A/20-HETE pathway increases pathological neovascularization in the tumor microenvironment (TME); (**3**) The CYP4A/20-HETE pathway induces the expression of HIF1a, VEGF, MMP2, MMP9, and other factors to increase migration, invasion, and metastasis. HET0016, a selective inhibitor of 20-HETE in the CYP4A pathway, decreases the metastatic potential of tumor cells, normalizes the blood flow, and controls abnormal neovascularization. The red boundary defines the tumor-associated vascular structure.

The first evidence pointing towards the role of the CYP4A/20-HETE axis in angiogenesis was pointed out by Sa et al. in their study, where they reported that FGF-2-mediated activation of cytosolic phospholipase A2 is responsible for AA production and CYP4A stimulation in endothelial cells [134]. Recent studies have shown that the CYP4A/F-20-HETE and VEGF pathways have a positive feedback regulation in circulating EPCs. Both hypoxia and VEGF induced expression of the CY4A11 gene and protein in EPCs, and 20-HETE and VEGF had a synergistic effect on EPC proliferation. Moreover, 20-HETE induced the expression of Very Late Antigen-4 (VLA-4) and CXCR4 in EPCs, thereby promoting their role in neovascularization, and targeting the 20-HETE pathway attenuated EPC-induced angiogenesis in a Matrigel plug angiogenesis assay [114]. 20-HETE offers a survival advantage to bovine pulmonary artery endothelial cells by activating the pro-survival PI3-kinase and Akt pathways, NADPH oxidase activation, and NADPH oxidase-derived superoxide, and thereby protects these cells from undergoing apoptosis [135]. There is a dynamic interplay between the tumor cells and the tumor stroma in regulating tumor growth, invasion, and metastasis, as shown in Figure 2. A bi-directional synergism comes into play to meet the metabolic and nutrient demands of the tumors and also to counter therapeutic resistance in the face of insult by chemotherapy and antiangiogenic therapy (AAT).

Our laboratory has shown that using HET0016, a 20-HETE inhibitor, decreased the level of several pro-angiogenic factors in a mouse model of TNBC, thereby affecting tumor growth [45]. T47D and BT-474 human BC cells overexpressing CYP4Z1 enhanced proliferation, migration, and tube formation of human umbilical vein endothelial cells (HUVECs), and promoted angiogenesis in the zebrafish embryo and chorioallantoic membrane of the chick embryo [136]. CYP4A1 expression in U251 glioma cells induced hyperproliferation both in vitro and in vivo, possibly due to the production of 20-HETE [117]. The CYP4A/20-HETE axis significantly increased tumor weight, microvessel density (MVD), and lung metastasis by upregulating VEGF and MMP in non-small cell lung cancer [137]. Many studies designed to understand tumor growth, the development of resistance to therapies, and metastasis have focused on tumor neovascularization as a primary target. A novel neovascularization mechanism that has gained widespread popularity amidst controversy is vascular mimicry [130,138,139]. Studies from our laboratory have demonstrated the efficacy of targeting the CYP4A/20-HETE axis in controlling vascular mimicry-dependent AAT resistance [120,122]. In light of the aforementioned evidence, studies of the CYP4A/20-HETE axis in mediating tumor growth, invasion, and metastasis serve to be an exciting domain of investigation.

Reports on the emerging role of the CYP4A/20-HETE axis in immune regulatory myeloid cells are building up. For the very first time, our laboratory has recently shown that pharmacological targeting of the CYP4A/20-HETE axis through HET0016 decreased g-MDSCs in the metastatic niche [44]. The growth factors and cytokines released by g-MDSCs can inhibit an effective T-cell response and promote the growth of disseminated tumor cells [37,140,141]. Chen et al. in a similar context, have shown that TAMs overexpressing a CYP4A10 variant can increase pre-metastatic niche formation and pulmonary metastasis [142]. Similarly, a novel flavonoid FLA-16, through inhibiting CYP4A pathways, normalized the tumor vasculature and improved survival. This was accompanied by the decreased secretion of 20-HETE, VEGF, and transforming growth factor beta (TGF-β) in TAMs and EPCs [143]. Altogether, CYP4A in TAMs is crucial for the tumor-dependent macrophage phenotype shift, and its inhibition by HET0016 or FLA-16 decreased tumor-associated phenotypes [142,143]. Moreover, these reports strongly suggest a critical role of the myeloid cell-produced 20-HETE metabolite in tumor growth and metastasis. In the future, more experimental investigations are needed to explore its role in other stromal cell types such as heterogeneous myeloid subsets, e.g., MDSCs and T cell subsets, which display a profound role in tumor and metastatic microenvironments (shown schematically in Figure 2). Since the TME is modeled around the availability of neovascular structures to meet the nutrient and metabolic demands of the tumor, the following section will highlight the role of the 20-HETE pathway in tumor and tumor-associated stromal cells with a special emphasis on endothelial cells.

## 6. HET0016 as a Novel Therapeutic Agent in Treatment of Metastasis

Currently, there is a dearth of studies investigating the CYP4A/20-HETE axis and its involvement in tumor growth and metastasis in patients. Increased levels of 12-HETE and 20-HETE were found in patients with prostate cancer and with myeloid leukemia [52,144]. In fact, various human cancer cells show the upregulation of 20-HETE-producing enzymes of CYP4A/F families including BC, colon and ovary cancer, and melanoma [142,145]. Our laboratory has extensively employed HET0016, a selective 20-HETE synthesis inhibitor, as a treatment to reduce the hyperproliferation of glioma [121,146,147] and BC cells [44,45].

Recently, we have shown that the growth of human glioblastoma was dwindled by an intravenous (IV) formulation of HET0016. We optimized the route of administration of the drug by making a novel IV formulation of HET0016 with 2-hydroxypropyl β cyclodextrin (HPβCD) to enhance bioavailability (resulting in a seven-fold higher level in plasma and 3.6-fold higher level in the tumor in the first hour compared to treatment via an intraperitoneal route) and to deliver an effective dose of the drug to the tumor site with reduced off-target effects and rapid clearance. We saw significantly reduced tumor growth with the IV HET0016 treatment in athymic nude rats that were orthotopically implanted with U251 cells. Similar growth inhibition was observed in the syngeneic GL261 GBM immunocompetent mouse model. Using magnetic resonance imaging (MRI), we evaluated the vascular kinetics in the TME, which showed that the delayed IV HET0016-treated animals have significantly lower v_p_ (blood plasma pool), v_e_ (extracellular space or interstitial volume), and K^trans^ (forward permeability transfer constant), and increased and normalized blood flow compared to that of the corresponding vehicle-treated groups. We observed a reduced expression of markers of cell proliferation (Ki67) and neovascularization (laminin, MVD, and αSMA), downregulation of pro-angiogenic proteins such as VE-cadherin (vascular endothelial cadherin-vasculogenesis), bFGF (basic fibroblast growth factor), IL-8 (chemokine CXCL8), SDF-1α (stromal cell-derived factor-1), and MCP-1 (a CCL2 ligand), and increased expression of anti-angiogenic proteins such as Tie-2, angiostatin, and angiopoietin-2/Tie-1 in the IV HET0016 treatment group. We determined the expression of different proteins by western blot and confirmed that HET0016 treatment decreases the level of markers of cellular proliferation (pERK), survival, migration, invasion (pAKT), inflammation (COX-1/2, p-NFκB), and angiogenesis (HIF-1α, EGFR, VEGF, and MMP2). Furthermore, we observed significantly improved survival in patient-derived xenograft (PDX) tumor models of GBM811 and HF2303 with HET0016 treatment alone or in combination with temozolomide (TMZ) in irradiated animals. Overall survival was prolonged to 26 weeks after combined treatment with HET0016 plus TMZ and radiation, while control animals survived for only 10 weeks in the GBM811 model while survival was prolonged to 26 weeks vs. 17 weeks for the irradiated control in the PDX model of HF2303 [121].

**Figure 3:**
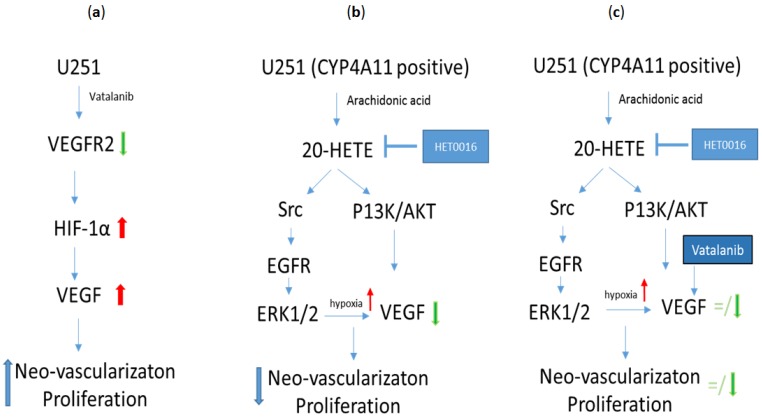
A possible mode of action of HET0016 in relation to growth factor pathways. (**a**) Treatment with vatalanib causes a decrease in expression of vascular endothelial growth factor receptor 2 (VEGFR2), but increases the expression of hypoxia-inducible factor 1 α (HIF-1α) and VEGF, which will cause increased neovascularization and tumor growth; (**b**) When HET0016 alone is used, VEGF expression is decreased through different signaling pathways, which will cause decreased neovascularization and tumor growth; (**c**) When HET0016 and vatalanib are used together some of the effects of vatalanib (increased VEGF, increased neovascularization and tumor growth) can be attenuated. Data obtained from Shankar et al. [46].

In one of our studies to understand the role of HET0016 in controlling metastasis, we have shown that HET0016 decreases migration and invasion in the metastatic TNBC cell line from both human (MDA-MB-231) and mouse (4T1) models in vitro. We also showed that IV HET0016 treatment reduces primary tumor growth and lung metastasis in 4T1 bearing immunocompetent Balb/c mice. Other studies have similarly observed a diminished expression of pro-inflammatory cytokines such as EGF, Fas, SDF-1α, IL-1β, IL-4, IL-17A, MMP-2, and MMP-9, which led us to investigate the downstream signaling mechanisms that could be affected by HET0016 [137,142,148,149,150]. The PI3K/Akt and MAPK signaling pathways are thought to be climacteric to regulate proliferation, invasion, angiogenesis, and metastasis ability [151,152,153]. Previously, it was found that 20-HETE is involved in activation of ERK1/2 and PI3K/Akt in endothelial cells [116] and also alters cell growth in U251 human gliomas by a mechanism that initially involves activation of the ERK1/2 pathway [117]. A schematic of the signaling pathway presented by Shankar et al. [46] from our research group summarizes the possible therapeutic actions of HET0016 in Figure 3. Yu et al. also showed that CYP ω-hydroxylase overexpression enhanced the lung metastasis of A549 cells in the nude mouse by upregulating VEGF and MMP-9 expression via the PI3K and ERK1/2 signaling pathway [137]. Our results showed reduced protein levels of pAKT, total AKT, pERK1/2, and pNFκB in lungs of animals treated with HET0016 compared to 4T1-bearing control mice. In recent years, many studies have shown the pivotal role of MDSCs in downregulating anti-tumor immunity and promoting tumor growth and metastasis [154,155,156]. We reported a novel role of HET0016 in impeding metastasis by decreasing the g-MDSCs polarization in the metastatic site [44].

## 7. Conclusions

Considering the evidence provided by studies from our and other research groups, the CYP4A/20-HETE axis has a multi-faceted role in promoting tumor growth and metastasis. This axis has also been highly activated in myeloid cells mediating immunosuppression in the TME. The tumor stroma consisting of tumor-associated endothelial cells proliferate and lay down neovascular structures induced by 20-HETE production. However, the CYP4A/20-HETE axis has been a neglected pathway in the development of novel therapeutics. We have successfully employed HET0016 in controlling glioma and breast tumor growth and metastasis. Therefore, novel therapeutics targeting the CYP4A/20-HETE axis should gain considerable importance in translational medicine, either as monotherapy or in combination with established chemotherapeutic and radiotherapeutic approaches.

## Figures and Tables

**Table 1:** Breast cancer subtypes classified according to immunohistochemical characterization. The details of prevalence, prognosis, and treatment of each subtype are presented. Data were obtained from the *Susan G. Komen Foundation* website ^1^ and the literature.

Subtypes	Molecular Characterization	Prevalence	Prognosis	Treatment
Luminal A	Estrogen receptor (ER)-positive, Progesterone receptor (PR)-positive or negative, Human epidermal growth factor receptor 2 (HER2)-negative	30–70% [2,3,4,5,6,7]	Best prognosis, high survival rates, and low recurrence rates [3,4,5,8]	Treatment for these tumors often includes chemotherapy and anti-hormone therapy
Luminal B	ER-positive, PR-positive or negative, HER2-positive	10–20% [2,3,4,5,6,7] Luminal B tumors are often diagnosed at a younger age than luminal A tumors [7,8,9]	Luminal B tumors tend to have factors that lead to a poorer prognosis, compared to luminal A tumors, including poorer tumor grade, larger tumor size and lymph node-positivity [3,4,5,8,9,10,11] Patients with luminal B tumors tend to have fairly high survival rates, although not as high as those with luminal A tumors [4,8]	The treatment for luminal B tumors includes anti-hormone therapy, anti-HER2 therapies and radiation, depending on tumor grade and lymph nodes status
HER2-enriched	ER-negative, PR-negative, HER2-positive	5–15% [3,5,7] HER2-type tumors may be diagnosed at a younger age than luminal A and luminal B tumors [8]	HER2-type tumors tend to have lymph node-positivity and poorer tumor grade [3,4,5,8,10]	HER2-type breast cancers can be treated with anti-HER2 drugs such as trastuzumab (Herceptin), lapatinib, capecitabine. Before these drugs were available, HER2-type tumors had a fairly poor prognosis [3,12]
Basal-like or Triple-negative breast cancer	ER-negative, PR-negative, HER2-negative	15–20% [2,3,4,5,6,7] These tumors tend to occur more often in younger women [5,9]	Triple-negative/basal-like tumors are often aggressive and have a poorer prognosis compared to ER-positive subtypes (luminal A and luminal B tumors) [3,5]	Triple-negative tumors can be treated successfully with chemotherapy and radiation, depending on tumor grade, lymph nodes status and disease stage

^1^ https://ww5.komen.org/BreastCancer/SubtypesofBreastCancer.html.

**Table 2:** CYP ω-hydroxylases that produce 20-HETE in mice, rats, rabbits, and humans. Data have been obtained from Roman [48].

Species	20-HETE Production
Mouse	CYP4A10; CYP4A12a; CYP4A12b; CYP4A14
Rat	CYP4A1; CYP4A2; CYP4A3
Rabbit	CYP4A4; CYP4A6; CYP4A7
Human	CYP4A11; CYP4A22; CYP4F2; CYP4F3

## Abbreviations

20-HETE	20-Hydroxy-eicosatetraenoic acid
4T1	Triple negative metastatic murine breast cancer cell line
ALX/FPR2	Lipoxin A4 receptor/formyl peptide receptor
AA	Arachidonic acid
AAT	Antiangiogenic therapy
AKT	Protein kinase B
AMPK	5′ AMP-activated protein kinase
BC	Breast cancer
BM	Brain metastasis
BSO	Buthionine sulfoximine
BT-474	Breast cancer luminal B subtype cell line
COX	Cyclooxygenase enzyme
CXCR4	C-X-C chemokine receptor type 4 also known as fusion or CD184;
CYP	Cytochrome P450
CYP4A	Cytochrome P450, family 4, subfamily A
CYP4F	Cytochrome P450, family 4, subfamily F
DHETs	Dihydroxy-eicosatrienoic acids
EES	Extravascular extracellular space
EETs	Epoxy-eicosatrienoic acids
EGF	Epidermal growth factor
EGFR	Epidermal growth factor receptor
sEH	Epoxide hydrolase
EPCs	Endothelial progenitor cells
ER	Estrogen receptor
ERK	Extracellular signal-regulated kinases
Fas	Fas ligand or CD95 ligand
FGF-2	Basic fibroblast growth factor 2
FLA-16	Novel flavonoid
GBM	Glioblastoma
GBM811	Glioblastoma-derived from patient
GL261	Murine glioblastoma cell line
GM-CSF	Granulocyte-macrophage colony-stimulating factor
GSH	Glutathione
GPx	Glutathione peroxidase
HER2	Human epidermal growth factor receptor2
HET0016	*N*-hydroxy-*N*′-(4-butyl-2 methyl phenyl) formamidine
HF2303	Glioblastoma-derived from patient
HIF-1α	Hypoxia-inducible factor 1 α
HO	Hepoxillin
HPβCD	2-Hydroxypropyl β-cyclodextrin
HPETE	Hydroperoxy-eicosatetraenoic acid
HUVEC	Human umbilical vein endothelial cells
IL	Interleukin
IV	Intravenous
Ki67	Proliferation marker
Ktrans	Forward permeability transfer constant
LOX	Lipoxygenase
LT	Leukotriene
LX	Lipoxin
MAPK	Mitogen-Activated Protein Kinase
MCP-1	Monocyte Chemoattractant Protein-1
MDA-MB-231	Triple negative metastatic human breast cancer cell line
MDSCs	Myeloid-derived suppressor cells
MMPs	Matrix metalloproteinases
MRI	Magnetic resonance imaging
MVD	Microvessel density
NADPH	Nicotinamide adenine dinucleotide phosphate
NFκB	Nuclear factor kappa-light-chain-enhancer of activated B cells
eNOS	Endothelial nitric oxide synthase
PDX	Patient-derived xenograft
PET	Polyethylene terephthalate
PG	Prostaglandin
PI3K	Phosphatidylinositol-3-kinases
PLA2	Phospholipase A2
PPARs	Peroxisome proliferator-activated receptors
PR	Progesterone receptor
ROS	Reactive oxygen species
SCF	Stem cell factor
SDF-1α	Stromal cell-derived factor 1 α
STAT1	Signal transducer and activator of transcription 1
T2DM	Type 2 diabetes mellitus
T47D	Breast cancer luminal A subtype cell line
TAMs	Tumor-associated macrophages
TGF-β	Transforming growth factor β 1
Tie-2	Transmembrane tyrosine-protein kinase receptor
TME	Tumor microenvironment
TMZ	Temozolomide
TNBC	Triple-negative breast cancer
TNFα	Tumor necrosis factor α
U251	Human glioblastoma cell line
ve	extracellular space or interstitial volume
VE-cadherin	Vascular endothelial cadherin
VEGF	Vascular endothelial growth factor
VLA-4	Very late antigen-4
vp	Blood plasma pool
α-SMA	α-smooth muscle actin
